# Mindful with your baby for mothers of infants with (parental) stress in a non-clinical setting: a wait-list controlled pilot trial

**DOI:** 10.1186/s12884-022-04640-z

**Published:** 2022-04-07

**Authors:** Eva Sophie Potharst, Irena Veringa-Skiba, Esther van Broekhuizen, Susan Maria Bögels

**Affiliations:** 1 Research Institute of Child Development and Education, University of Amsterdam, Nieuwe Achtergracht 127, 1018 WS Amsterdam, The Netherlands; 2 Academic Outpatient (child and adolescent) Treatment Center, UvA Minds, University of Amsterdam, Banstraat 29, 1071 JW Amsterdam, The Netherlands; 3 Developmental Psychology, University of Amsterdam, Nieuwe Achtergracht 129-B, 1018 WS Amsterdam, The Netherlands

**Keywords:** Mindfulness, Mindful parenting, Mothers, Infants, Parental stress, Intervention

## Abstract

**Background:**

Because of the far-reaching negative consequences of high levels of (parental) stress for the mother, infant, the mother-infant relationship, and family functioning, psychological support for young mothers is important. Mindful with Your Baby is a mindfulness-based intervention, originally developed and evaluated in a clinical population of mothers with mental health issues and/or babies with regulation problems. The current pilot examines whether Mindful with Your Baby for mothers with symptoms of (parental) stress offered in a non-clinical setting is also effective and acceptable.

**Methods:**

In this pilot waitlist-controlled trial, 17 mothers with infants (2–15 months) admitted themselves for a Mindful with Your Baby training in a non-clinical setting because of (parental) stress. Mindful with Your Baby was offered in groups of three to six mother-infant dyads and consisted of eight weekly 2-h sessions. Participants completed questionnaires on symptoms of parental stress, general stress, depression, anxiety, mindfulness and self-compassion at 8-week waitlist, pretest, posttest and 8-week follow-up.

**Results:**

There were no training drop-outs, attendance rate was 92.5%, and the training was evaluated positively: all mothers (100%) felt they got something of lasting importance as a result of taking the training, and reported becoming more conscious as a parent, and 93% reported changing their lifestyle or parenting as a result of the training. Multilevel analyses showed no significant changes between waitlist and pretest. At posttest, a significant improvement occurred in all outcome measures compared to pretest, of moderate to large effect sizes. At follow-up, a significant improvement was seen compared to pretest in all outcomes except anxiety compared to pretest, of small to moderate effect sizes.

**Conclusions:**

Mindful with Your Baby appears an acceptable and effective intervention for mothers with a baby who experience (parental) stress but who have not been referred to specialized mental health care. A low threshold access to Mindful with Your Baby in non-clinical settings could provide a timely and positive interference in (parental) stress.

## Introduction

High parental stress in the first year postpartum has negative consequences for the parent-infant relationship [1], and for infant development [2]. The postnatal period plays an important role in the intergenerational transmission of vulnerability to stress [3]. One of the pathways through which this intergenerational transmission may take place, is through the impact of maternal stress on the infant neuroendocrine changes [4]. Cortisol levels play a role in stress reactivity and altered cortisol levels are predictive of later psychopathology [4]. In case of elevated levels of stress in mothers a timely, effective, and evidence-based intervention is important [5]. A systemic review of systematic reviews of interventions to improve maternal mental health and well-being of mothers of infants concluded that most studies focused on intervention for postpartum depression, while little attention was given to interventions focusing on postpartum stress [5].

In the last decades, there has been an increase in evidence regarding the value of mindfulness interventions for people with stress-related complaints [6]. A mindfulness training is a group intervention in which people learn to pay attention in a particular way: on purpose, in the present moment, and non-judgmentally [7]. It has been shown that mindfulness intervention is not only effective to decrease stress in general, but also parental stress [8]. A mindful parenting (MP) training is an application of mindfulness aimed at improving parenting by improving the quality of parental attention, increasing awareness of parental stress, and decreasing stress reactivity and the intergenerational transmission of stress vulnerability [8]. MP may thus be a suitable intervention for mothers with postnatal elevated levels of parental stress. An adjusted MP training for mothers with a baby has been developed for a clinical population: Mindful with your baby (MwyB) [9]. This training was offered to mothers and babies who had been referred to an outpatient mental health clinic because of elevated levels of stress or mental health problems of the mother, infant regulation problems, or problems in the mother–child interaction.

A study on the effectiveness of MwyB in mental health care in which 44 mothers-infant dyads participated, showed that between pretest and posttest, significant improvements was found in maternal self-reported mindfulness, self-compassion, mindful parenting, well-being, parental confidence, and responsivity and hostility in relation to her baby [9]. Improvements were maintained at both the 8-week and 1-year follow-up. However, this study did not include a control group or waitlist condition, which limited the conclusions that could be drawn from the results [9]. A second study on the effectiveness of MwyB (and Mindful with your Toddler) did include a waitlist condition and made use of observational instruments [10]. In this study, 36 mother-baby dyads were included, and an additional 14 mother-toddler dyads. On the basis of mother–child video observations, maternal sensitivity and acceptance, maternal mind-mindedness, and emotional communications between the mother and infant, were coded. In the time period prior to the training, between the waitlist assessment and pretest assessment, no improvements were found. When posttest was compared with pretest, significant improvements were found in maternal acceptance and mind-mindedness, and child responsiveness.

Studies evaluating MwyB for mothers with infants who do suffer from (parental) stress, but have not been referred for mental health care, are lacking. However, also mothers who do not suffer from mental health problems may feel a need for support in their stress-regulation abilities [11]. The transition to motherhood brings about changes in many aspects of a women’s life. Not only does she need to adjust to all the new developmental tasks that are involved in mothering, such as the regulation of the infant’s sleeping and eating pattern, and the forming of a bond with the infant, but also other roles and relationships in her life change in this time period [12]. The demands that are associated with becoming a mother pose a risk for a high level of stress, which can have negative influences for the parenting quality [13]. Parental stress is for example associated with overreactive parenting, and decreased parental sensitivity [1, 13, 14]. It is important to offer mothers who experience elevated levels of (parental) stress an intervention that is more broadly available than in mental health care only. MwyB, offered in a low threshold (non-clinical) setting, may be a suitable intervention for mothers who experience (parental) stress.

In the current study, we aimed to evaluate MwyB for mothers of infants who admitted themselves to the training because of symptoms of (parental) stress. We were especially interested in the question whether MwyB was effective and acceptable when offered in a non-clinical setting, for mothers who were not referred but self-selected. We used a longitudinal design, with waitlist, pretest, posttest, and 2-month to study the treatment effects. We hypothesized that MwyB would be effective in decreasing symptoms of parental stress, stress, depression, anxiety, and increasing mindfulness, and self-compassion, and that the effects would be maintained up to eight weeks after the training ended.

## Methods

### Participants

Mothers who experienced symptoms of parental stress admitted themselves for the current study. Inclusion in the study was based on the mothers’ subjective experience of parental stress. Further, participants with previous acute psychotic episode or diagnosed psychotic disorder, current suicidal risk, current substance use of dependency, borderline personality disorder or current trauma unrelated to childbirth were excluded from the study. Seventeen mothers with 2- to 15- months old infants (*M*_*age*_ = 8.20 months; *SD* = 3.63; nine boys (52.9%); nine firstborns (52.9%)) participated in the current study. Most mothers lived with the father of the baby (88.2%) and two (11.8%) lived alone with their baby. Fourteen mothers (82.4%) were born in The Netherlands and three (17.6%) had a different country of birth. Nine mothers (52.9%) obtained a university degree, seven (41.2%) a high vocational education degree, and one (5.9%) a high-school diploma. Eight mothers (47.1%) were working a part-time job, three (17.6%) were on sick leave, two (11.8%) were working a full-time job, two (11.8%) were stay-at-home mothers, one mother (5.9%) was a student and one (5.9%) was on maternity leave. Seven mothers (41.2%) received individual psychological support between the birth of their baby and the start of the training, and one of these mothers and her baby (5.9%) also received an intervention for excessively crying babies.

### Procedure

**Fig. 1:**
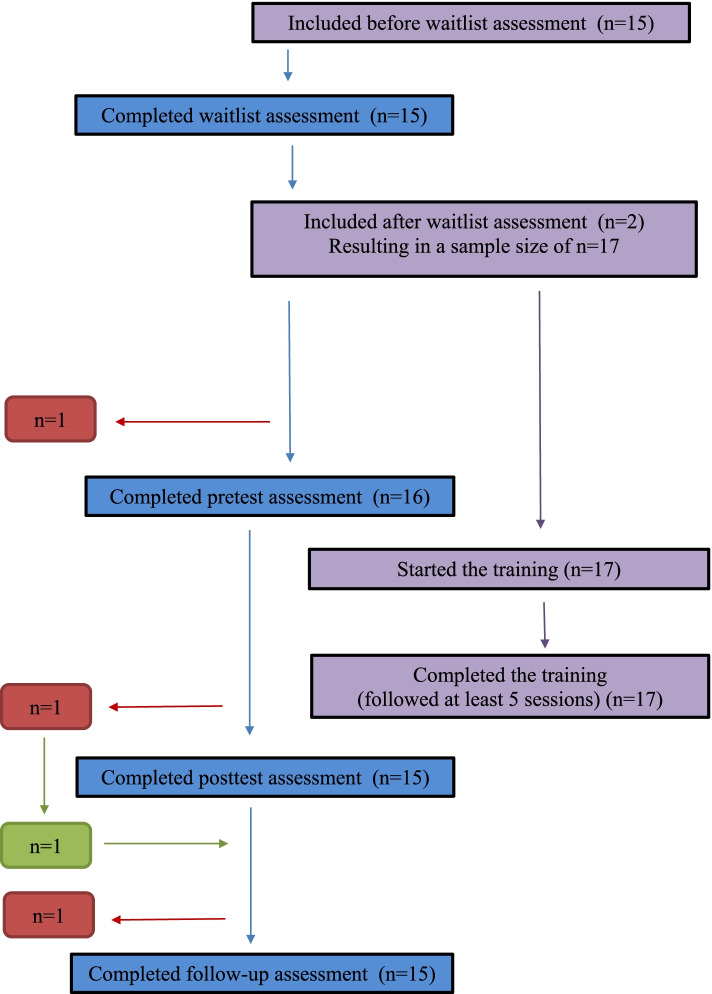
Flow diagram

This study used a quasi-experimental design. The ethical committee of the of the Faculty of Social and Behavioral Sciences of the University of Amsterdam approved the study (2015-CDE-4632). Participants were recruited in various ways. About half (52.9%) of the mothers in the current study had already participated in a randomized control trial (RCT) that investigated the effectiveness of Mindfulness-Based Childbirth and Parenting (MBCP) training versus enhanced care-as-usual for pregnant women and their partners recruited because of women’s high fear of childbirth [15]. All 141 participants from that RCT were invited to participate in the current study in the first year after giving birth. Six mothers from the experimental and three mothers from the control group admitted themselves for the current study. The other participants were recruited through Facebook groups for pregnant women, and mothers with babies, and groups focused on giving birth. In the invitation (email or Facebook), mothers were asked to participate in a study on stress reduction in mothers with babies by means of a mindfulness training. In the invitation, it was also explained that the training could be followed for free, but that participants would be asked to complete questionnaires. Those who were interested could download a more elaborate flyer, and admit themselves to the training and study. After admitting themselves to the study, participants were called by the trainer, to check inclusion criteria: a subjective experience of (parental) stress, and the ability to speak and read Dutch. Exclusion criteria were: a current unsafe situation for the baby, and maternal psychosis or suicidality. None of the mothers that admitted themselves were excluded. Participants gave informed consent online, before completing the first set of online questionnaires using forced responses via Qualtrics software (Qualtrics, Provo, UT, USA). A waitlist assessment was administered 8 weeks prior to the start of the training, to control for the effects of time, assessment and other intervention. Two participants were admitted to the study close to the start of a new training, and did therefore not complete the waitlist assessment. Waitlist assessment was thus completed by 15 mothers (88.2% of the complete sample). The pretest assessment took place in the week before the start of the training, posttest the week after the end of the training and follow-up eight weeks after the end of the training, respectively. The response rate was 94.1% at pretest, and 88.2% at posttest and follow-up. A flow diagram of participation in the study and the training can be found in Fig. 1.

### Intervention

The MwyB training is an adaptation of the MP training [16] and builds upon the Infant Mental Health approach [17] by including the babies in most of the sessions. MwyB consists of 8 weekly 2-h sessions, and a follow-up session 8 weeks after the last session. It is adapted to the presence of the babies (in 7 of the 9 sessions), and the themes that play a role for most mothers with a baby. The sessions consist of formal meditation, inquiry, a discussion of home practices, mindful parenting (visualization) practices, psychoeducation, and watching meditations with attention for the baby. Although the presence of the babies may sometimes be distracting, it also offers many learning opportunities. Stressful situations with the baby may arise during the sessions, which offers the possibility to practice on the spot with taking breathing space meditations with the support of the trainer, which may support generalization of what is being learned to the home situation. Because the babies are present, mothers can also practice with staying present with them in an accepting way, but also with paying attention to their own experience while caring for their baby. Exceptions are the first and the fifth session, that are attended by the mothers only. In the first session mindfulness and mindful parenting are introduced, and in the fifth session self-compassion. Details of the training can be found in Table 1. While in the study by Potharst et al. [9], a mental health psychologist/mindfulness/MP trainer plus an infant mental health specialist provided the training, in this study the training was provided by a licensed midwife/mindfulness/MP/MBCP trainer plus an assistant (a master student Psychology or Pedagogics). The main role of the assistant was to ensure both physical and emotional safety of the babies during the meditations in which mothers closed their eyes. Four groups of 3 to 6 dyads were given MwyB between March 2016 and December 2017.

**Table 1:** Themes and practices of the Mindful with your Baby training per session

Title of the session	Themes	Practices	Home practices	Readings
1. Becoming aware of the automatic pilot (session for mothers only)	Introduction to mindfulness and mindful parenting Automatic pilot Automatic stress responses	Intention meditation Raison practice Visualization on a stressful situation	Formal: bodyscan Informal: Mindful routine activity, Mindful eating Mindful parenting: Feeding the baby mindfully	The transition of becoming a parent Attention, mindfulness, and awareness
2. Mindfully observing your baby (session for mothers and babies together)	Beginner’s mind Friendliness	Breathing meditation Visualization on attitude towards yourself as a parent in stressful moments Watching meditation with attention for the baby	Formal: Bodyscan / breathing meditation Informal: Mindfulness diary for pleasant experiences Mindful parenting: Mindful routine activity with baby	Signals form your baby The seven attitudinal qualities
3. Creating space for yourself (session for mothers and babies together)	Becoming aware of own needs Becoming aware of what gives energy The role of bodily sensations	Meditation with attention for breath and body 3-min breathing space Practice ‘What do I need?’ Watching meditation with attention for the baby	Formal: Meditation with attention for breath and body Informal: 3-min breathing space Mindful parenting: Mindfulness diary for stressful experiences	Supporting your baby’s autonomy Being at home in your body
4. Responding sensitively to your baby (session for mothers and babies together)	Sensitive responsivity Parental overreactivity Regulation	Meditation with attention for breath and sounds Parent–child breathing space Watching meditation with attention for the baby	Formal: Meditation with attention for breath and sounds Informal: text messages in group to remind each other of taking a breathing space Mindful parenting: Parent–child breathing space in stressful moments	Regulation Stress and empathy
5. Taking care of yourself in difficult moments (session for mothers only)	The effect of thoughts Self-compassion	Meditation with attention for breath and thoughts Walking meditation Zen Koan Self-compassion meditation	Formal: Meditation with attention for breath and thoughts, Walking meditation Informal: Self-compassion meditation Mindful parenting: Parent–child breathing space in specific recurring stressful moment	Comforting your crying baby Thoughts
6. Closeness and distance (session for mothers and babies together)	Feelings of closeness and distance Repair after moments of distance	Metta meditation Visualization on dealing with feelings of distance Watching meditation with attention for the baby	Formal: Metta meditation Informal: Mindful walking Mindful parenting: Becoming aware of moments of closeness and distance, and consciously repairing the contact after moments of distance	The Circle of Security Feeling heard and seen
7. Dealing with expectations (session for mothers and babies together)	Expectations Finding your way in parenting Boundaries	Meditation with open attention Mindful movement Visualization on boundaries Watching meditation with attention for the baby	Formal: Meditation with open attention, Mindful movement Informal: Reflection on mindfulness in daily life and learning process Mindful parenting: Becoming aware of expectations and boundaries	Boundaries Dealing with unpredictability
8. Mindful parenting: trial and error (session for mothers and babies together)	Learning process Inspiration and support on mindfulness path	Sitting with a difficulty Practice looking back and ahead Gratitude exercise	Following own meditation plan	
Follow-up session (session for mothers and babies together)	Catching up on the 8 main themes of the training	Mountain meditation Evaluation of meditation plan Practice on experience of the baby A wish for yourself and your baby	Following adjusted meditation plan	

### Measures

#### Parental stress

Parental stress was assessed with the short form of the Dutch Parenting Stress Index (PSI) [18], based on the American PSI [19]. Two of the 25 items were eliminated as they did not seem applicable for mothers with a baby. Parents rated each item on a 6-point Likert scale, ranging from 1 to 6. Scores can be interpreted as very low, low, below the average, average, above the average, high, and very high [18]. The Dutch PSI possesses good reliability [18]. In the current study, Crohnbach’s alpha was 0.94 at pretest.

#### Symptoms of stress, depression, and anxiety

Mothers’ symptoms of stress, depression, and anxiety were assessed by a short form of the Depression Anxiety Stress Scales (DASS-21) [20]. Each subscale consists of 7 items which are scored on a 4-point scale ranging from 0 to 3. The DASS-21 possesses adequate reliability and validity [21]. DASS-21 subscale scores range from 0 to 21 and can be interpreted with regard to severity as normal, mild, moderate, severe, and extremely severe scores [20]. In this study, pretest Crohnbach’s alphas were 0.91, 0.86, and 0.72 for the stress, depression, and anxiety scale, respectively.

#### Mindfulness

Mindfulness was assessed using the short form [22] of the Dutch version [23] of the five-facet mindfulness questionnaire (FFMQ) [24]. The 24 items were scored on a 5-point Likert scale ranging from 1 to 5. The psychometric properties of the original scale and short form were good [22, 23]. Here, Crohnbach’s alpha was 0.88 at pretest.

#### Self-compassion

To measure self-compassion, we used the 3-item Self-Compassion Scale (SCS-3) [25]. The items were scored on a 7-point Likert scale ranging from 1 to 7. The internal consistency of this 3-item scale was found to be 0.74, and the correlation with the total score of the 12-item short form of the SCS 0.90 [25]. In this study, the Crohnbach’s alpha at pretest was 0.80.

#### Acceptability

Acceptability was assessed by an evaluation questionnaire at posttest (see Table 1 for the topics that were covered by the evaluation questionnaire). We used an adapted version of the stress reduction program evaluation, developed at the Center for Mindfulness of the University of Massachusetts Medical School (see [16]). Session attendance rate, reported in the evaluation questionnaire, was calculated by dividing the number of attended sessions by the total number of sessions.

### Data analyses

Data analyses Inspection of variable distributions indicated sufficient normality; skewness and kurtosis of all variables were <|2|. Hypotheses on the effects of the training on all outcomes were tested with multilevel regression models that are known to accommodate dependence between observations, and missing data [26, 27]. Therefore, all 17 cases, also those with one or more missing measurements, could be included in the analyses. The structure of the multilevel models consisted of the repeated measurements of the outcomes across the measurement points (at waitlist, pretest, posttest, and follow-up) nested within the participating mothers. Measurement occasions were dummy coded with pretest scores as reference. Scores on all outcomes were standardized across assessments, so that parameter estimates can be interpreted as effect sizes. Effects were regarded as significant when *p* < 0.05.

## Results

All mothers and babies who started the intervention completed it (*n* = 17). Treatment completion was defined as following at least 5 out of the 8 sessions. The average session attendance rate was calculated based on the number of sessions that the mothers who completed the posttest evaluation form reported (*n* = 15). The average attendance rate was 92.5% for the eight weekly sessions. Acceptability of the MwyB training was high, as shown by the positive evaluations at posttest (see Table 2). Mean scores (SD) on all outcome measures at waitlist, pretest, posttest, and follow-up are displayed in Table 3. At waitlist and pretest, mean levels of parental stress can be interpreted as above average [18], of general stress as extremely severe, and of depression and anxiety as moderate, as compared to norm groups of the general population [20].

**Table 2:** Evaluation of the Mindful with your baby training at posttest (*n* = 15)

Question	Yes	No		
Do you feel you got something of lasting value as a result of taking this training?	15 (100%)	0 (0%)		
Have you made any changes in lifestyle or parenting as a result of the training?	14 (93.3%)	1 (6.7%)		
Did you become more ‘conscious’ in parenting?	15 (100%)	0 (0%)		
Is it your intention to continue practicing the formal meditations?	15 (100%)	0 (0%)		
Do you have the intention to continue practicing mindful parenting?	15 (100%)	0 (0%)		
	Never	1–2 times	3–4 times	5–7 times
How often did you practice the formal meditations at home during the training usually?	0 (0%)	6 (40%)	6 (40%)	3 (20%)
Has there been change as a result of the training in:	Clear	Some	No	Negative
Knowing how to take better care of yourself?	3 (20%)	8 (53.3%)	4 (26.7%)	0 (0%)
Actually taking better care of yourself?	2 (13.3%)	8 (53.3%)	4 (29.4%)	0 (0%)
The frequency of parental stress?	3 (20%)	9 (60%)	3 (20%)	0 (0%)
The intensity of parenting stress or frustration?	2 (13.3%)	8 (53.3%)	5 (33.3%)	0 (0%)
Being content with the relationship with your child?	2 (13.3%)	11 (73.3%)	2 (13.3%)	0 (0%)
The confidence you have in yourself as a mother?	4 (26.7%)	8 (53.3%)	2 (13.3%)	1 (6.7%)
Feeling hopeful as a mother?	2 (13.3%)	8 (53.3%)	5 (33.3%)	0 (0%)
Dealing with emotions while taking care of or parenting your child?	3 (20%)	11 (73.3%)	1 (6.7%)	0 (0%)
Awareness of what is stressful in your life?	5 (33.3%)	9 (60%)	1 (6.7%)	0 (0%)
Awareness of stressful parenting situations at the time they are happening?	5 (33.3%)	8 (53.3%)	2 (23.3%)	0 (0%)
The ability to handle stressful parenting situations appropriately?	3 (20%)	9 (60%)	3 (20%)	0 (0%)
How many, of the total of eight sessions, have you been present?	7.4 (.8)			
	Likert scale ranging from 1 (not important) to 10 (enormously important)			
How important has the training been for you?	7.33 (1.63)			

Data are presented as *n* (%) or mean (standard deviation)

**Table 3:** Outcomes of all measures at waitlist, pretest, posttest, and follow-up

Outcome variable	Waitlist		Pretest		Posttest		Follow-up	
	*n*	*M (SD)*	*n*	*M (SD)*	*n*	*M (SD)*	*n*	*M (SD)*
Parental stress (PSI-SF)	15	2.80 (.79)	16	2.52 (.87)	15	2.17 (.63)	15	2.19 (.66)
Number (%) scoring above average or higher	10 (66.7%)		7 (43.8%)		3 (20%)		5 (33%)	
Stress, Depression, and anxiety (DASS-21)								
Stress	15	20.00 (7.52)	16	17.88 (8.69)	15	12.67 (6.75)	15	13.20 (6.67)
Number (%) scoring (extremely) severe	13 (86.7%)		12 (75.0%)		6 (40.0%)		6 (40.0%)	
Depression	15	9.87 (6.82)	16	8.50 (7.06)	15	4.93 (5.85)	15	4.40 (5.14)
Number (%) scoring (extremely) severe	5 (33.3%)		5 (31.3%)		4 (26.7%)		3 (20.0%)	
Anxiety	15	6.67 (5.05)	16	5.63 (5.23)	15	2.93 (2.91)	15	3.60 (2.95)
Number (%) scoring (extremely) severe	6 (40.0%)		5 (31.0%)		2 (13.3%)		1 (6.7%)	
Mindfulness (FFMQ-SF)	15	2.96 (.39)	16	2.86 (.50)	14	3.73 (.95)	15	3.12 (.53)
Self-compassion (SCS-3)	15	3.33 (1.23)	16	3.42 (1.30)	14	4.10 (1.04)	15	3.96 (1.05)

Data are presented as mean (standard deviation) or numbers (percentages). The scores presented are mean sum scores for the DASS-21 scales (scale range 0–21) and mean item scores (1- 6 for the NOSI-K, 1–5 for the FFMQ-SF, 1–7 and for the SCS-3)

The Mindful with your baby training took place between pretest and posttest

*PSI-SF* short form of the Dutch Parenting Stress Index *DASS-21* 21-item version of the Depression Anxiety Stress Scales, *FFMQ-SF* short form of the Five Facets Mindfulness Questionnaire, *SCS-3* 3-item version of the Self-Compassion Scale

Results of multilevel models of treatment outcome predicted by measurement occasion are displayed in Table 4. No significant differences were seen in outcomes between waitlist and pretest. At posttest, a significant improvement was seen in all outcome measures compared to pretest. The decrease in maternal symptoms of parental stress, stress, depression, and anxiety were of moderate effect size, and the increase in mindfulness and self-compassion were of moderate and large effect size, respectively. At 8-week follow-up, the improvements were still significant compared to pretest, except for symptoms of anxiety. The decrease in stress and depression, and the increase in mindfulness was of moderate effect size. The decrease in parental stress and increase in self-compassion was of small to moderate effect size.

**Table 4:** Results of multilevel regression models of treatment outcome predicted by measurement occasion (deviations from pre-test)

	Intercept		Waitlist		Posttest		Follow-up	
	*β (SE)*	*F*	*β (SE)*	*F*	*β (SE)*	*F*	*β (SE)*	*F*
Parental Stress (PSI-SF)	.13 (.26)	.26	.34 (.17)	3.98^a^	-.47 (.17)	7.70^b^	-.44 (.18)	6.09^b^
Stress, depression, and anxiety (DASS-21)								
Stress	.28 (.26)	1.16	.23 (.24)	.94	-.58 (.26)	5.13^b^	-.62 (.26)	5.93^b^
Depression	.41 (.30)	1.85	.02 (.16)	.02	-.64 (.19)	10.86^c^	-.71 (.17)	18.18^c^
Anxiety	.26 (.28)	.88	.32 (.15)	4.61^a^	-.64 (.22)	8.41^b^	-.42 (.19)	4.69^a^
Mindfulness (FFMQ-SF)	-.37 (.24)	2.35	.20 (.24)	.65	.95 (.21)	19.82^c^	.58 (.21)	7.51^b^
Self-compassion (SCS-3)	-.22 (.26)	.73	.01 (.22)	< .01	.58 (.22)	6.78^b^	.47 (.20)	5.38^b^

Data are presented as parameter estimates (and standard errors) and *F* values of multilevel regression models

*β* = Parameter estimate, which can be interpreted as Cohen’s *d* effect size of change

*PSI-SF* short form of the Dutch Parenting Stress Index, *DASS-21,* 21-item version of the Depression Anxiety Stress Scales, *FFMQ-SF* short form of the Five Facets Mindfulness Questionnaire, *SCS-3* 3-item version of the Self-Compassion Scale

^a^*p* < .10. ^b^*p* < .05. ^c^*p* < .01

A post hoc power analysis was conducted using the software G*Power [28]. For the calculation, a repeated measures MANOVA and an alpha error probability of 0.05 was selected. Furthermore, we used the means or the correlations between waitlist and pretest, pretest and posttest, and posttest and follow-up, and the effect sizes found between pretest and posttest were included in the calculation for each outcome measure. Power was shown to be 0.94 for parental stress, 0.70, 0.99, and 0.74 for symptoms stress, depression, and anxiety, respectively, > 0.99 for mindfulness and 0.80 for self-compassion.

## Discussion

In the current study, we aimed to evaluate MwyB, for mothers with symptoms of (parental) stress. We were especially interested in the question whether this training was acceptable and effective when not offered in a specialized mental health setting (as in [9, 10]), but instead in a non-clinical setting such as midwifery care. If so, this would offer possibilities regarding the accessibility of the training for a larger group of women with symptoms of (parental) stress, who are not (yet) in need for specialized mental health care. This study showed that the training was acceptable in this form, as shown by the high attendance rates, zero dropout, and positive evaluations. Also, results offered evidence that MwyB is effective in decreasing symptoms of parental stress, stress, depression, and anxiety, and in increasing mindfulness and self-compassion.

Pretest scores showed participating mothers’ elevated levels of symptomatology, especially regarding stress in general. Pretest scores differed only slightly with those measured in an earlier study on MwyB offered in specialized mental health care [9]. In the current study, mothers showed slightly better functioning with respect to parental stress and self-compassion (about one third of a standard deviation difference), but worse regarding mindfulness (about half a standard deviation) compared to the clinical population of a previous MwyB study [9]. Results imply that self-selection for the training works well, as participants had indeed elevated levels of symptomatology and a need for support.

No significant improvements between waitlist and pretest were shown, indicating that the improvements between pretest and posttest can be attributed to the training rather than the effects of assessment and time alone. Effects between waitlist and pretest were especially important to examine, as about one third of the mothers received other forms of psychological support in the period between giving birth and the start of the training.

As expected, both stress in general and parental stress decreased after the training. This is in line with the idea that mindfulness and mindful parenting training affects both for intrapersonal and interpersonal problems [29]. We also found improvement regarding symptoms of depression and anxiety, and mindfulness and self-compassion, just as in the study on MwyB in specialized mental health care [9]. The effects in the study of Potharst et al. [9] were somewhat smaller at posttest for parental stress (non-significant), anxiety and depression (almost moderate), and mindfulness (moderate) than in de current study, but comparable at follow-up. The effect on self-compassion on the other hand was somewhat larger at posttest and follow-up (large) than in the current study. This implies that MwyB seems to be (at least) as effective in a non-clinical as in a clinical setting. Our findings are in line with a study on a Mindful Parenting training for parents of older children, where we found similar improvements in a clinical versus a non-clinical setting [30].

The question is what the mechanisms of change were in the current study. The significant improvement in mindfulness and self-compassion suggests that these factors may have played a role in the improvement in mothers’ mental health problems and parental stress [31]. Whether indeed mindfulness and self-compassion were mediators of these improvements could not be examined in the current study because of limited power.

Even though part of the participating mothers (35%) had already received a mindfulness training during their pregnancy, all mothers (100%) reacted positively to the questions whether they felt they had gotten something of lasting value, whether they had become more conscious in parenting, and almost all mothers (93%) to the question whether they had made changes in their lifestyle or parenting. This may imply that practicing mindfulness in the presence of the baby has added value to practicing mindfulness when pregnant, and that MwyB supports women in integrating mindfulness in this new phase of life.

Several limitations of the current study need to be taken into account. First of all, there was no control group receiving another intervention. This makes it impossible to conclude anything about the effectiveness of the training compared to other interventions. Second, the sample consisted only of mothers, was self-selected, and not clearly defined because we did not make use of a screening instrument. Third, only maternal self-report measures were included. A future study should ideally be a randomized study, comparing Mindful with your baby with another intervention, include a larger sample of both fathers and mothers, and include more objective outcome measures.

## Conclusions

MwyB appears an acceptable intervention and effective in decreasing (parental) stress and symptoms of depression and anxiety, and improving mindfulness and self-compassion for mothers with a baby who experience (parental) stress but have not been referred to specialized mental health. A low threshold access to MwyB in non-clinical settings such as midwifery care could timely interfere in (parental) stress and reach more mothers in need. In this way, midwives could take more responsibility in caring for the mental well-being of new families.

## Data Availability

The datasets used and/or analysed during the current study are available from the corresponding author on reasonable request.

## Abbreviations

MP: Mindful parenting
MwyB: Mindful with your baby
RCT: Randomized control trial
MBCP: Mindfulness-Based Childbirth and Parenting
PSI: Parenting Stress Index
DASS: Depression Anxiety Stress Scales
FFMQ: Five-facet mindfulness questionnaire
SCS: Self-Compassion Scale
